# Effectiveness of functional hand splinting and the cognitive orientation to occupational performance (CO-OP) approach in children with cerebral palsy and brain injury: two randomised controlled trial protocols

**DOI:** 10.1186/1471-2377-14-144

**Published:** 2014-07-15

**Authors:** Michelle Jackman, Iona Novak, Natasha Lannin

**Affiliations:** 1 University of Notre Dame, School of Medicine, Sydney, Australia; 2 Department of Occupational Therapy, John Hunter Children’s Hospital, Locked Bag 1, HRMC, Newcastle, NSW 3210, Australia; 3 Cerebral Palsy Alliance Research Institute, Sydney, Australia; 4 Department of Occupational Therapy, La Trobe University, Faculty of Health Sciences, Melbourne, Australia; 5 Occupational Therapy Department, Alfred Health, Melbourne, Australia

**Keywords:** Cerebral palsy, Splints, Motor learning, Hand, Randomised controlled trial

## Abstract

**Background:**

Cerebral palsy (CP) and brain injury (BI) are common conditions that have devastating effects on a child’s ability to use their hands. Hand splinting and task-specific training are two interventions that are often used to address deficits in upper limb skills, both in isolation or concurrently. The aim of this paper is to describe the method to be used to conduct two randomised controlled trials (RCT) investigating (a) the immediate effect of functional hand splints, and (b) the effect of functional hand splints used concurrently with task-specific training compared to functional hand splints alone, and to task-specific training alone in children with CP and BI. The Cognitive Orientation to Occupational Performance (CO-OP) approach will be the task-specific training approach used.

**Methods/Design:**

Two concurrent trials; a two group, parallel design, RCT with a sample size of 30 participants (15 per group); and a three group, parallel design, assessor blinded, RCT with a sample size of 45 participants (15 per group). Inclusion criteria: age 4-15 years; diagnosis of CP or BI; Manual Abilities Classification System (MACS) level I – IV; hand function goals; impaired hand function; the cognitive, language and behavioural ability to participate in CO-OP. Participants will be randomly allocated to one of 3 groups; (1) functional hand splint only (n=15); (2) functional hand splint combined with task-specific training (n=15); (3) task-specific training only (n=15). Allocation concealment will be achieved using sequentially numbered, sealed opaque envelopes opened by an off-site officer after baseline measures. Treatment will be provided for a period of 2 weeks, with outcome measures taken at baseline, 1 hour after randomisation, 2 weeks and 10 weeks. The functional hand splint will be a wrist cock-up splint (+/- thumb support or supination strap). Task-specific training will involve 10 sessions of CO-OP provided in a group of 2-4 children. Primary outcome measures will be the Canadian Occupational Performance Measure (COPM) and the Goal Attainment Scale (GAS). Analysis will be conducted on an intention-to-treat basis.

**Discussion:**

This paper outlines the protocol for two randomised controlled trials investigating functional hand splints and CO-OP for children with CP and BI.

## Background

Cerebral palsy and brain injury are common neurological conditions in childhood [1,2] that can have devastating effects on a child’s ability to use their hands [3]. Hand splints and task-specific training are widely used interventions to help improve the hand function of children with cerebral palsy and brain injury. Splinting to improve hand function has been under-researched, whereas task-specific training to improve hand function (using various approaches) is well supported by high quality evidence [4-9]. Little is understood about whether the two treatment approaches have an augmentative effect when used simultaneously.

Cerebral palsy is a group of disorders of the development of movement and posture, causing activity limitations that are attributed to non-progressive disturbances that occurred in the developing fetal or infant brain [1]. Brain injury in children is a brain insult acquired in the post neonatal period, although there is no defined lower age limit [1]. Children with cerebral palsy and brain injury experience similar difficulties in regard to hand function [10,11], and similar treatment options, including hand splinting and task-specific training, are utilised in both conditions. The evidence base regarding therapeutic interventions for children with cerebral palsy is comparatively larger than in other diagnostic groups. Despite brain injury being recognised as one of the leading causes of long-term disability in children and young adults, there is a recognised lack of evidence for this population [12]. Cerebral palsy and brain injury have been previously grouped together for research purposes [10], and given the common symptoms and management strategies, it is logical these diagnostic groups may be combined in therapeutic intervention research.

### Hand splinting

Splinting to facilitate hand function is a widely practiced intervention in the treatment of children with neurological conditions, although there is little reliable evidence to support this approach [10,13,14]. A ‘hand splint’ may be a brace, orthosis, cast, tape or any external device applied to one or more joints of the upper limb. Two theoretical approaches have historically underpinned the rationale for splinting; these are the neurophysiological and the biomechanical approaches. In the neurophysiological approach it is thought that splints might work by inhibiting the reflexive contraction of muscles [15], although biological and clinical evidence questions the validity of this splinting theory [15]. In the biomechanical approach splints are thought to reposition the hand into a biomechanically advantageous position for optimising hand function [15]. The biomechanical theoretical underpinning is yet to be supported by evidence. There are two primary types of upper limb splints, whose purpose may be defined according to the International Classification of Functioning, Disability and Health (ICF) model [16]. There are splints that are prescribed for the purpose of bringing about changes in the body function and structure domain of the ICF, such as a serial cast with the aim of lengthening muscles, or a ‘resting splint’ designed to stretch muscles [17]. These splints generally interfere with voluntary hand function, due to their physical form, and are therefore generally worn at night, or prescribed for short periods of time. The second category of hand splints are ‘functional splints’, which aim to address deficits in the activity and participation domain of the ICF [13]. Functional splints are prescribed for use during activities to directly improve task performance, such as a wrist cock-up splint designed to stabilise the wrist during tasks such as handwriting or cutlery use at mealtimes. A functional hand splint is designed to improve use of the hand, and in doing so, takes into consideration not only the underlying biomechanical components on the hand, but also directly addresses the goals and preferences of the person wearing the splint [17].

Preliminary evidence suggests that functional hand splints may have an immediate positive effect on hand function [18], and provide a supplementary effect to goal directed training [19]. Whilst splints may provide a very small clinical effect, it is unclear whether this leads to any improvement in function [13], and evidence suggests this immediate improvement may not be maintained beyond the splint-wearing period [13]. There is no reliable evidence to support the use of hand splints as a therapeutic modality in isolation of other intervention approaches [13]. In addition to splinting to reposition the hand, the patient may also be trained to use their hand in a functional way using a task-specific training approach.

### Task-specific training

Task-specific upper limb training is a term used to encompass upper limb interventions which involve active use of the limb during task practice. Other terms, such as motor training, task training, activity-focused motor interventions and repetitive task practice are used in the literature to refer to this group of interventions, which includes constraint-induced movement therapy (CIMT), bimanual training and goal-directed training. Task-specific training interventions are underpinned by principles of motor learning and neuroplasticity, that is, repetitious active motor sequences that aim to induce changes in the neural pathways [20]. Task-specific training interventions that focus on tasks that are engaging, meaningful and challenging have been shown to maximise motor learning and neural plasticity [21].

Over the past 10 years, a strong evidence base has emerged to support task-specific interventions for children with cerebral palsy [6-9,22,23] and although each intervention is different, we now know that there are common factors among these interventions that are likely to lead to positive outcomes. These factors include:

1. Goal-directed.

2. Repetitive.

3. Task or activity specific (in which the child actively, rather than passively participates).

4. High dose (although optimal dose is unclear especially for those with brain injury or non-hemiplegic cerebral palsy).

The focus of occupational therapy intervention for children with cerebral palsy and brain injury is evolving. Task specificity and dose are two factors influencing this change. Task specificity is consistent with a top-down approach to intervention, in which it is recognised that the whole task needs to be practiced in order for improvements in that task to be achieved. Previously, bottom-up approaches to intervention were common, in which the underlying factors contributing to a child’s ability to carry out a task were addressed with the assumption that this would lead to improved task performance. If viewed in line with the ICF model, top-down approaches directly address deficits in the ‘activity and participation’ domain, whilst bottom-up approaches address deficits in the ‘body function and structure’ domain. Evidence suggests that changes in the body function and structure domain should not be assumed to influence changes in the activity and participation domain of the ICF [24], supporting the benefits of task-specific interventions. Dose, or amount, of therapeutic intervention, is another factor that is changing from traditional models of therapy [25]. There is strong evidence to support the superior efficacy of high dose upper limb interventions [5,7,8,26], provided over a short period, whereas there is limited evidence to support the effectiveness of more traditional therapy, provided in a smaller total dose on a regular basis (for example, a block of therapy provided on a fortnightly basis). Research is currently being undertaken to compare the effectiveness of these two different approaches in the cerebral palsy population [27].

The recent body of evidence supporting upper limb task-specific training interventions [5,7,8] is primarily in the unilateral cerebral palsy population. The evidence to support task-specific training in brain injury, and other typographies of cerebral palsy, is limited, although theorists expect similar results are possible. There is little published evidence regarding task-specific training, utilising the Cognitive Orientation to Occupational Performance (CO-OP) approach in this population.

#### The cognitive orientation to occupational performance (CO-OP) approach

The CO-OP approach is a child-centred, goal-focussed approach that combines task-specific training with learning theories [28,29]. The CO-OP approach is a problem-solving process designed to enable children with difficulties carrying out motor-based tasks to reach their functional goals, enabling them to perform the activities they need to, want to, or are expected to do in everyday life [29]. There is an important distinction between CO-OP and other upper limb task-specific training approaches. Whilst other approaches are based around motor learning theories of repetitive task practice, CO-OP differs in that it is centred first around the child learning the global cognitive strategy of goal-plan-do-check. It is after this cognitive strategy is understood that the child then applies the strategy to motor-based tasks in the repetitious action consistent with other task-specific training approaches. One of the key features of the cognitive theory underlying the CO-OP approach are the benefits of the child undergoing the process of ‘guided discovery’ in which the child plans, attempts, reviews and discovers the strategies to effectively perform motor based tasks, rather than therapists or parents identifying solutions for the child, or prompting the child to perform the task in a certain way [29,30]. Through discovering the solutions to motor based problems themselves, learning theories suggest that the child will have a greater chance of retaining the learnt skill [29]. Whilst in other approaches goal setting may be led by caregivers, one of the prerequisites for participation in CO-OP is that children set their own goals and therefore have the internal motivation to practice and succeed at the CO-OP tasks. Motivation in therapy is a factor gaining recognition in upper limb intervention research. Recently, upper limb task-specific training interventions have utilised novel approaches to therapy, through magic [31], pirate [23] or circus [27] themes to improve motivation. Motivation for participation and practice in CO-OP is addressed only through goal setting and thus inclusion of individually meaningful task practice [29,32].

The CO-OP approach was developed for children with developmental coordination disorder (DCD) and there is strong evidence to support the effectiveness of CO-OP in the DCD population [33]. Preliminary evidence has also shown promising results in children with Aspergers syndrome [34,35] and Pervasive Developmental Disorder [36]. Initial investigations regarding the use of CO-OP in the paediatric brain injury population showed that goals were achieved and maintained, however that participants had difficulty applying the global problem-solving strategy to other goals beyond those targeted in therapy, although some of the general strategies identified could be applied to other tasks [37]. Parent involvement was recognised as an important factor in success [37]. Initial investigation regarding the use of CO-OP for children with cerebral palsy has indicated positive outcomes [38], however these findings are yet to be published. Whilst there does not yet exist extensive evidence regarding the use of CO-OP in cerebral palsy and brain injury, the theoretical foundations underpinning the CO-OP approach are consistent with interventions proven to be effective in this population [7,25,39,40].

Evidence indicates that positive outcomes can be achieved through a smaller dose of treatment using CO-OP, compared to alternative forms of task-specific training [33]. For example, doses of CIMT and bimanual training in high quality studies have involved greater than 60 hours of treatment [5,7,8] compared to the 12 hours of treatment required in the CO-OP approach [29]. Another benefit of the CO-OP approach is the generalisation, or ‘carry-over’ effect of skills learnt during intervention. This suggests that children may continue to benefit from CO-OP by applying strategies learnt to future goals and activities the child wishes to participate in beyond the treatment timeframe. This was one factor that may not have occurred for children with a brain injury who undertook CO-OP [37]. If comparable benefits can be achieved through the use of CO-OP, via a much smaller dose of therapy, and with the potential carry-over benefits beyond the goals of treatment, CO-OP may provide a cost-effective upper limb intervention option as the child progresses through adolescence and into adulthood. The CO-OP approach is a therapeutic intervention that can be utilised to address upper limb difficulties in all typographies of cerebral palsy and brain injury, and is not limited to the unilateral neurological population.

#### Functional hand splints combined with task-specific training

Differing views exist in clinical practice about the relationship between upper limb task-specific training and functional hand splints [15,17]. Some assert that a functional splint will improve movement and hand use immediately when the child dons the splint within everyday hand function activities [18]. Others assert that it takes longer to train the child to use the hand efficiently and that the splint should be worn during this training period to help shape the desired hand movements required for function [15,41]. The use of a splint to biomechanically reposition a joint, whilst potentially improving function, may also produce the unwanted effect of inhibiting joint movement and therefore inhibiting muscle activity [15]. Given the theories underpinning task-specific training treatment approaches, the use of a functional splint to support or immobilise a limb may in fact inhibit the patient’s opportunity for motor re-learning [15]. The effect of a functional splint, compared to or combined with task-specific training, on hand function is yet to be studied using rigorous methodologies.

### Objectives

The aim of this paper is to describe the method used to conduct two randomised controlled trials investigating the effect of functional hand splints combined with task-specific training, utilising the CO-OP approach.

#### Objective 1

The primary objective of this study is to investigate whether functional hand splints combined with task-specific training leads to greater improvements in goal achievement and hand function in children with cerebral palsy and brain injury, compared to functional hand splints alone or task-specific training alone.

##### Hypothesis 1

Children with cerebral palsy and brain injury who receive a functional hand splint combined with task-specific training will achieve comparable improvements on the Canadian Occupational Performance Measure (COPM), Goal Attainment Scale (GAS) and Box and Block Test (BBT) scores when compared to children who receive task-specific training alone.

#### Objective 2

The second objective of this study aims to investigate whether a functional hand splint provided to a child with cerebral palsy or brain injury leads to an immediate improvement on the BBT.

##### Hypothesis 2

Children with cerebral palsy and brain injury provided with a customised functional hand splint to wear will have immediately higher BBT scores compared to children with cerebral palsy and brain injury that do not wear a hand splint.

#### Objective 3

The third objective of this study is to explore the use of the CO-OP approach for children with cerebral palsy and brain injury.

##### Hypothesis 3

Children with cerebral palsy and brain injury who participate in CO-OP therapy will achieve clinically significant changes in goal achievement, according to the COPM and GAS.

## Methods

### Trial design

**Figure 1:**
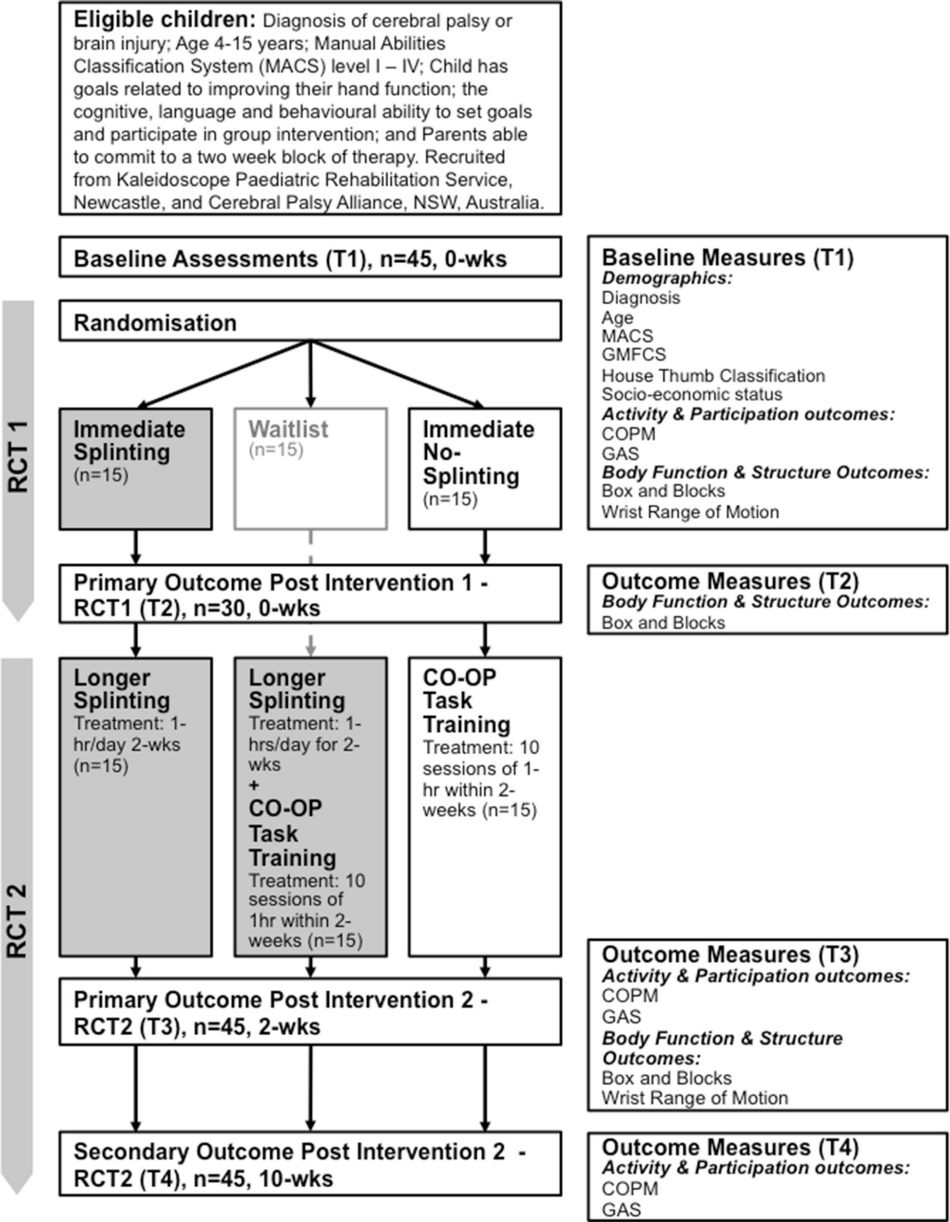
Flow chart of RCT1 and RCT2 according to CONSORT guidelines.

The study will involve two randomised controlled trials (RCT) undertaken concurrently with both using a parallel design RCT to address the research objectives. Participants enrolled in the study will participate in both RCTs, depending on group allocation. This process is described in Figure 1.

RCT1 will investigate the immediate effect of a functional hand splint on hand function using a two group, parallel design randomised controlled trial with a sample size of 30 participants (15 participants per group). RCT2 will investigate the effect of functional hand splints combined with task-specific training (utilising the CO-OP approach), compared to functional hand splints alone or task-specific training alone in children with cerebral palsy and brain injury. RCT2 will involve a three group, parallel design, assessor blinded, randomised controlled trial with a sample size of 45 participants (15 participants per group).

### Participants

Inclusion criteria:

• Diagnosis of cerebral palsy or brain injury (minimum 12 months post injury).

• Age 4-15 years.

• Manual Abilities Classification System (MACS) level I – IV.

• Impaired hand function as a result of the neurological condition.

• Goals related to improving hand function.

• Sufficient language, cognitive and behavioural skills to set goals, interact with the therapist and participate within a group context (according to CO-OP guidelines [29]).

• Parents able to commit to a two week block of therapy.

Exclusion criteria

• Known allergy to thermoplastic splinting material.

• Impaired hand function resulting from secondary condition (eg. Fracture or burn).

• Significant intellectual or language impairment (according to CO-OP guidelines [29]).

• Current treatment not compatible with the study.

Children will not be excluded from the study if they have received upper limb injections of Botulinum Toxin A, although this data will be collected prior to the intervention and taken into consideration during statistical analyses.

### Ethical considerations and registration

This trial has been approved by the Human Research Ethics Committees of Hunter New England Area Health Service (HREC/11/HNE/410 and 11/11/16/4.03), The University of Notre Dame Australia (012042S) and The Cerebral Palsy Alliance (2012-02-01). The trial was registered with the Australian New Zealand Clinical Trials Registry (ACTRN12613000690752).

### Recruitment

Forty five children aged 4 – 15 years, with a diagnosis of cerebral palsy or brain injury will be recruited from across New South Wales and Victoria, Australia. Recruitment of potential participants will occur through the Kaleidoscope Paediatric Rehabilitation Service (Newcastle), Monash Children’s Hospital (Melbourne) and the Cerebral Palsy Alliance (Newcastle and Sydney regions). Face to face and email contact will be made with local paediatric therapy services working with the paediatric cerebral palsy and brain injury population. Where possible, contact will also made with community support groups involved with the study population. Potential participants will be screened by the chief investigator, then assessed for eligibility prior to being enrolled in the study. Informed consent will be obtained from all parents/guardians and assent obtained from children over the age of 8 years.

### Sample size

A total of 45 children will participate in the study. Sample size calculations for RCT2 were estimated using data from a 3 group RCT involving children with cerebral palsy [9], based on multiple regression analysis of data using the Canadian Occupational Performance Measure (COPM) accounting for 2 covariates (group allocation and age). Based on an anticipated effect size of 0.9, power analysis for the 3 group RCT determined that 15 participants per group would give an 80% probability of detecting a clinically significant effect of 2 points on the 10 point COPM scale, with statistical significance set at <0.05.

The BBT will be utilised as the primary outcome for RCT1 (the immediate effect of a splint on hand function). The authors felt that the COPM was not an appropriate immediate outcome measure as goal achievement is unlikely to occur immediately, and as such may not provide a true representation of the capacity for a hand splint to have an immediate positive effect on hand function. The BBT test is thought to be a sensitive gross test of hand function. There are studies utilising the BBT in the unilateral cerebral palsy population [42], however there was no previously published homogeneous sample applicable to our study on which to base a sample estimate using the BBT. As such, we were unable to determine an appropriate sample size, and based the sample size according to that set by RCT2. As such, a total of 30 participants will participate in the two group RCT1 (15 participants per group).

### Randomisation

#### Sequence generation

Random sequence generation was achieved using computer random number generation.

#### Allocation concealment mechanism

Sequentially numbered, sealed opaque envelopes, opened by an offsite officer not involved in the study, will be used to allocate group. After baseline measures are taken, the assessor will contact the officer via phone to obtain group allocation. This will ensure assessors and participants are blinded to group allocation during baseline measurement.

#### Blinding

Participants and assessors will be blinded to group allocation at baseline measurement, as randomisation will occur after baseline measures are taken. Due to the nature of the therapeutic intervention, participants and treating therapists are unable to be blinded to group allocation. To reduce participant bias, participants will not be informed of the study hypotheses. In RCT 1, due to the fact that the splint will be worn during the box and blocks test, assessors will not be able to be blinded to treatment group. For RCT2, assessors will blinded to group allocation and order of assessment. Measures that will be taken to ensure that participants do not disclose group allocation to assessors will include (a) participants being informed of the importance of blinding during the consent process and when follow up assessments are booked, (b) assessors reminding participants at the beginning of the assessment not to discuss the intervention received, and (c) assessors not reviewing participant log books.

Data regarding hand function with the splint off, as well as the splint on (for those allocated to a splint group) were seen as important to collect. As having the splint on during assessment would lead to the assessor not being blinded to treatment group, all outcome measures will be taken and scored with the splint off (without disclosure of group allocation), following which the participant will be asked if they had a hand splint. If the participant has a splint, the box and blocks test will be repeated with the splint on. A different blinded assessor will complete outcome measures at each time point to ensure blinding.

Data will be entered by a person not involved in the study.

### Study procedures

Figure 1 depicts the study procedure. Informed consent will be obtained following screening for eligibility criteria. Baseline assessment, using the COPM, GAS, BBT and wrist range of motion will be completed within 3 weeks of treatment (T1). Following baseline assessment, participants will be randomly allocated to one of 3 treatment groups:

1. Functional hand splint alone.

2. Task-specific training (CO-OP) combined with functional hand splint.

3. Task-specific training (CO-OP) alone.

Participants allocated to a group involving a functional hand splint will have a customised hand splint fabricated immediately following randomisation. One hour following baseline assessment, the box and blocks test will be repeated with participants allocated to the functional hand splint alone (with splint on) or task-specific training alone (no splint) group (T2). This data will be used to investigate the immediate effect of a functional hand splint (RCT1). All participants will then undertake 2 weeks of treatment, according to group allocation. The functional hand splint alone group will be expected to wear the splint for 1 hour of daily practice of goals. Task-specific training will involve 10 daily one hour sessions of CO-OP conducted over 12 consecutive days. CO-OP will be undertaken within a group of 2-4 participants. Children allocated to the task-specific training (CO-OP) combined with functional hand splint will be expected to wear their hand splint at all times during the 10 sessions of CO-OP. Assessment at the end of the 2 weeks of treatment (T3) will involve the COPM, GAS, Box and blocks test and wrist range of motion. Log books of home practice will be completed by all participants. Participants will not be expected to continue practice of goals beyond the 2 week treatment timeframe. Participants will be asked to complete a log book of practice if they choose to continue to utilise the functional hand splint or practice goals using those strategies from CO-OP intervention. Follow up assessment, using the primary outcomes only (COPM and GAS) will occur at 10 weeks following baseline assessment (T4).

### Interventions

The total duration of the intervention will be 2 weeks, regardless of group allocation.

#### Functional splinting

The functional splint used in this study will be a wrist cock-up splint, with the addition of a thumb support or supination wrap if deemed appropriate for the individual child. The wrist splint will be made from thermoplastic alone or a combination of neoprene and thermoplastic. The decision to make a splint from thermoplastic alone, or a combination of thermoplastic and neoprene, will be made taking into consideration the level of support required at the wrist, the participant’s goals, and the participant’s aesthetic preference. Aesthetic preference was taken into consideration in order to improve compliance [43], and to remain consistent with considerations that would be applied in regular therapeutic situations. A thermoplastic wrist extension support on the palmer surface will be a consistent component of all splints. The wrist cock-up splint will aim to support the wrist in approximately 20 to 30 degrees of extension, however, where this amount of extension is unable to be achieved, the splint will be positioned in maximum wrist extension that is tolerable to the participant, whilst allowing for functional use of the hand. A thumb support will be included for participants whose thumb metacarpophalangeal is adducted, when the thumb position is deemed to impact on grasp and release or the child’s individual goal achievement. A supination strap will be added to the splint for children who are unable to actively supinate beyond neutral, and where supination is deemed an important prerequisite for the child’s identified goals.

#### The cognitive orientation to occupational performance (CO-OP)

Task-specific training will be carried out utilising the CO-OP approach. The standard CO-OP protocol comprises 12 sessions, with the initial session involving assessment and goal setting, subsequent 10 sessions focussed on teaching and utilising the global strategy goal-plan-do-check and the final session reviewing goals and encouraging carry-over of strategy use beyond the treatment period. For in depth details of CO-OP, the full protocol should be reviewed [29].

In the current study, general principles of the intervention will follow the CO-OP protocol, with some adaptations. During the screening process, parents will be provided with general information regarding the CO-OP approach and informed that a parent is expected to be present at all treatment sessions. The initial assessment will be an individual session in which each child will identify 3 goals related to hand function, nominating one goal that is most important to them. If participants are randomised to receive CO-OP, a more indepth discussion with parents regarding CO-OP will occur. CO-OP will be conducted in a group setting, for a total of 10 treatment sessions (5 times per week for 2 weeks). Groups will consist of 2-4 participants and each session will run for approximately 1 hour. A parent/carer will be required to be present at every session. The first group session will focus on educating parents regarding the goal-plan-do-check strategy, and introducing children to the strategy, through the use of a puppet – Captain Goal-Plan-Do-Check. Subsequent sessions will focus on the use of goal-plan-do-check for motor-based tasks, as a group at the beginning of each session, then individually with the assistance of parents throughout the session. The therapist will demonstrate the use of goal-plan-do-check and prompt parents to support children to utilise this strategy. Children will identify one goal that is most important to them, and, where possible, the whole group will work on this goal.

#### Therapeutic considerations

Group intervention was chosen for cost and time efficiency. The CO-OP approach can be utilised in a group format [29] and task-specific training intervention provided in a group setting has been shown to be effective in this population [5,44]. The benefits of group intervention may include improved motivation and participation [44]. The treating therapist will apply generalised therapeutic considerations during the groups, as would be applied in standard practice. These will include adapting the group structure, modifying tasks, providing further assistance to children/parents who require it and implementing behavioural management strategies.

#### Reliability training of assessors and treatment administrators

The treating therapist will be an experienced occupational therapist, who has undergone formal training regarding upper limb splinting and the use of the CO-OP approach, and has experience using both of these approaches in clinical practice. All assessors (experienced occupational therapists) will undergo training regarding standardised use of outcome measures. A trial manual has been developed to ensure consistency in administration of assessments.

### Measures

#### Demographic and classification measures

Demographic information will be collected from participants prior to baseline measurement. This will include the participant’s diagnosis, age, manual abilities classification system (MACS) level, gross motor function classification system (GMFCS) level, House thumb classification, cognition, behaviour, socioeconomic information and details regarding any current or recent upper limb interventions including occupational therapy, Botulinum Toxin A injections or surgery.

##### Manual abilities classification system (MACS)

The Manual Abilities Classification System (MACS) is a system used to describe how children with cerebral palsy use their hands to handle objects in everyday activities [45]. This classifies five levels of hand use on a continuum from I (Independent with age appropriate hand tasks, able to handle objects easily and successfully) to V (Child is dependent, unable to handle objects and severely limited ability to perform simple actions) [45]. Parents and the child will be actively involved in determining the child’s MACS level to describe how the child uses their hands on a daily basis, rather than describing the child’s maximum capacity to use their hands. Although not developed for children with brain injury, the MACS is an appropriate tool to enable a consistent classification of hand function in all participants of the present study, and as such will be used to describe hand function in participants with brain injury as well as those with cerebral palsy.

##### Gross motor function classification system (GMFCS)

The Gross Motor Functional Classification System (GMFCS) is a system used to describe a child’s functional gross motor abilities [46]. The GMFCS describes five levels of motor ability from I (Independent mobility, walks without limitations) to V (Dependent for mobility, transported in an attendant-propelled wheelchair) across 3 age groups: 4-6 years; 6-12 years and; 12-18years [46]. Within the clinical setting, the GMFCS may be used as a predictive tool and would not be appropriate for use for a child with a brain injury. In this study the GMFCS will be used as a descriptive tool to gain consistent baseline information regarding gross motor ability across all study participants, regardless of diagnosis of brain injury or cerebral palsy.

##### House thumb classification

The House Thumb Classification is a tool that describes common thumb deformity [47], and has been found to be a reliable descriptor in the cerebral palsy population [48]. The assessor identifies the most appropriate thumb classification from 4 visual options, based on the position of the child’s thumb during activity.

#### Outcome measures

Primary outcome measures will measure change in the activity and participation domain of the ICF, using the COPM and GAS, and secondary outcome measures will measure changes in the body function and structure domain of the ICF, using the BBT test and wrist range of motion, as shown in Table 1. Outcome measure timepoints are reflected in Figure 1.

**Table 1:** Classification of outcome measures according to the ICF model

**Outcome measures according to ICF domain**	
**Body function and structure**	**Activity and participation**
Wrist range of motion	COPM
Box & blocks test	GAS

#### Primary outcomes

##### The Canadian occupational performance measure (COPM)

The COPM is an individualised, child and family focussed tool that is used to measure change in goals that are meaningful to the individual [49]. The COPM measures change in the activities and participation domain of the ICF. The COPM is carried out as an interview, structured around the areas of self-care (dressing, toileting, feeding), leisure (hobbies/interests, sports) and productivity (school). The child and family identify areas of difficulty, prioritise these difficulties, then rate the child’s performance and satisfaction in those areas identified as most important to the child. It is the performance and satisfaction scores that are used to measure change. The 3 upper limb tasks identified as most important will be set as the goals for therapy. There is strong evidence to support the internal consistency reliability, content and construct validity and responsiveness of the COPM when used in paediatric rehabilitation [44,50]. The COPM has been shown to be responsive to change, with a 2 point improvement on performance scores recognised as clinically significant [49,51]. All participants, regardless of age, will be involved in the COPM process of identifying meaningful goals as is recognised as an important component of the CO-OP protocol [29].

##### The goal attainment scale (GAS)

The GAS is a measure of change of client-specific goals that may not be measurable through standardised assessments [50,52]. The GAS may offer children and families the opportunity to set a wider range of goals than those set using the COPM, particularly in regard to upper limb activities [50]. Following the administration of the COPM, 3 GAS goals related to upper limb function will be set. Goals will be formatted on a 5 point scale; -2 (current level of function); -1 (less than expected level of performance); 0 (expected level of performance), +1 (more than expected level of performance); +2 (much more than expected level of performance). Whilst being recognised as being responsive to change [50], there is still limited evidence supporting the reliability and validity of GAS for detecting clinically significant changes [52,53]. A change of 2 points on the GAS is generally recognised as significant (ie. a change from -2, current level of function to 0, expected level of performance) [53].

#### Secondary outcomes

##### The box & block test (BBT)

The BBT is a brief assessment of hand function, in which the participant is required to transfer as many blocks as possible from one box to another, with one hand, in 60 seconds [54]. Whilst this is a gross assessment of hand function, the BBT provides a standardised assessment of grasp and release, which are important components of hand function. Evidence supports the validity, test-retest reliability and interrater reliability [55] of the BBT [54]. Previous studies suggest that a change score of 4 blocks per minute over a 2 week treatment timeframe represents clinically significant change [55]. It is unknown whether repeating the BBT within a one hour timeframe (for RCT1) will have a practice effect.

##### Wrist joint range of motion

Active and passive joint range of motion of wrist extension (with fingers in a resting position) and Volkmann’s angle (with fingers in extension) will be measured. Measurement will be taken using a device to enable assessors to use a consistent amount of force when taking wrist measurements, given that goniometry measurement of wrist extension has been shown to have moderate interrater reliability [48]. Children will be seated at the edge of a table, with their forearm flat on the wrist device. The palm will be placed on the wrist device and 1kg of force applied to the wrist into extension. To decrease measurement error, a digital inclinometer will be used to verify the measurement (degrees), and a still photograph taken of the inclinometer reading. To measure active wrist extension, the child’s hand will be unstrapped from the device, with the forearm staying in same position as passive range of motion. The child will be asked to extend their wrist, and a demonstration of wrist extension with the fingers flexed will be given. Volkmann’s angle is a measure of digitial flexor tendon tightness [56]. That is, a measure of wrist extension with the fingers held in extension. This measurement will be taken with the child seated, with the forearm strapped into the device as detailed above. With the wrist in flexion, the fingers of the hand will be extended, then the wrist extended to its maximum range without compromising finger extension. As in the other measurements described above, a digital inclinometer will be used to measure range of motion.

### Adverse events

Adverse events, including pain, skin irritation and discomfort will be collected and recorded at each outcome measure timepoint. If adverse events relate to the use of the hand splint, the hand splint will be modified or use discontinued. If significant or unintended adverse events occur, parents will be encouraged to have the child reviewed by a medical practitioner.

### Statistical methods

Participant attributes will be analysed using descriptive and inferential statistics to assess baseline comparability among the 3 treatment groups. Comparison of differences between the 3 groups in the randomised controlled trial will be analysed using multiple regression analysis, with statistical significance set at p < 0.05. Statistical analysis in RCT1 will be conducted using 2 group comparisons on all participants, based on the BBT (T1 vs T2). Data from all randomised participants will be conducted on an intention to treat basis. Where outcome data are unavailable, previous scores will be carried over for analysis. Statistical analysis for RCT2 will be conducted at 2 weeks (T3) and 10 weeks (T4) compared to (T1) baseline scores, analysing between group differences. Primary analyses at T3 and T4 will be based on the COPM and GAS scores. Secondary analysis will use the same data analytical methods for scores on the BBT and range of motion for T3 only.

## Discussion

This paper outlines the methods that will be used to conduct two randomised controlled trials investigating (a) the immediate effect of functional hand splints, and (b) the relationship between upper limb task-specific training and functional hand splinting in regard to goal achievement in children with cerebral palsy and brain injury. It is anticipated that the results of this study will be disseminated through publication in peer-reviewed journals and at academic conferences.

### Consent

Written informed consent was obtained from the parents/carers of all participants for publication of this trial. A copy of the written consent is available for review by the Editor of this journal.

## Abbreviations

CP: Cerebral palsy; BI: Brain injury; RCT: Randomised controlled trial; CO-OP: Cognitive orientation to occupational performance; MACS: Manual abilities classification system; COPM: Canadian occupational performance measure; GAS: Goal attainment scale; BBT: Box and block test; ICF: International classification of functioning, disability and health; CIMT: Constraint-induced movement therapy; GMFCS: Gross motor function classification system.

## Competing interests

The authors declare no competing interests. There was no funding attached to this research at the time of protocol publication. This research is being funded by a National Health and Medical Research Council (NHMRC) scholarship grant (MJ, 1074570).

## Authors’ contributions

MJ (PhD candidate) designed the RCT and wrote the protocol. IN (supervisor) and NL (supervisor) designed the RCT, contributed to the content of and critically appraised this protocol. All authors have read and approved the final manuscript.

## Pre-publication history

The pre-publication history for this paper can be accessed here:

http://www.biomedcentral.com/1471-2377/14/144/prepub

## References

[B1] Rosenbaum P Paneth N Leviton A Goldstein M Bax M A report: the definition and classification of cerebral palsy April 2006 Dev Med Child Neurol 2007 49 8 14 17370477

[B2] Feigin VL Theadom A Barker-Collo S Starkey NJ McPherson K Kahan M Dowell A Brown P Parag V Kydd R Jones K Jones A Ameratunga S Incidence of traumatic brain injury in New Zealand: a population-based study Lancet Neurol 2013 12 1 53 64 23177532 10.1016/S1474-4422(12)70262-4

[B3] Arner M Eliasson AC Nicklasson S Sommerstein K Hägglund G Hand function in cerebral palsy: report of 367 children in a population-based longitudinal health care program J Hand Surg 2008 33 8 1337 1347 10.1016/j.jhsa.2008.02.032 18929198

[B4] Hoare Brian J Wasiak J Imms C Carey L Constraint-induced movement therapy in the treatment of the upper limb in children with hemiplegic cerebral palsy Cochrane Database Syst Rev 2007 2 CD004149 17443542 10.1002/14651858.CD004149.pub2

[B5] Gordon AM Hung Y-C Brandao M Ferre CL Kuo H-C Friel K Petra E Chinnan A Charles JR Bimanual training and constraint-induced movement therapy in children with hemiplegic cerebral palsy: a randomized trial Neurorehab Neural Re 2011 25 8 692 702 10.1177/1545968311402508 21700924

[B6] Novak I McIntyre S Morgan C Campbell L Dark L Morton N Stumbles E Wilson S-A Goldsmith S A systematic review of interventions for children with cerebral palsy: state of the evidence Dev Med Child Neurol 2013 55 10 885 910 23962350 10.1111/dmcn.12246

[B7] Hoare B Imms C Villanueva E Rawicki HB Matyas T Carey L Intensive therapy following upper limb botulinum toxin A injection in young children with unilateral cerebral palsy: a randomized trial Dev Med Child Neurol 2013 55 3 238 247 23236956 10.1111/dmcn.12054

[B8] Wallen M Ziviani J Evans R Naylor O Novak I Herbert R Modified constraint-induced therapy compared with standard occupational therapy for children with hemiplegic cerebral palsy: results of a randomised trial: occupational therapy Australia, 24th national conference and exhibition, 29 June - 1 July 2011 Aust Occup Ther J 2011 58 73 74

[B9] Novak I Cusick A Lannin N Occupational therapy home programs for cerebral palsy: double-blind, randomized, controlled trial Pediatrics 2009 124 4 e606 e614 19770175 10.1542/peds.2009-0288

[B10] Teplicky R Law M Russell D The effectiveness of casts, orthoses, and splints for children with neurological disorders Infant & Young Child: An Interdisciplinary J of Spec Care Prac 2002 15 1 42 50

[B11] Chevignard M Toure H Brugel DG Poirier J Laurent-Vannier A A comprehensive model of care for rehabilitation of children with acquired brain injuries Child: Care, Health Dev 2010 36 1 31 43 19438875 10.1111/j.1365-2214.2009.00949.x

[B12] Baxter P Acute and chronic management of traumatic brain injury Dev Med Child Neurol 2013 55 8 680 680 23834202 10.1111/dmcn.12196

[B13] Jackman M Novak I Lannin N Effectiveness of hand splints in children with cerebral palsy: a systematic review with meta-analysis Dev Med Child Neurol 2014 56 138 47 23848480 10.1111/dmcn.12205

[B14] Autti-Rämö I Suoranta J Anttila H Malmivaara A Mäkelä M Effectiveness of upper and lower limb casting and orthoses in children with cerebral palsy: an overview of review articles Am J Physical Med & Rehab/Assocof Acad Physiatrists 2006 85 1 89 103 10.1097/01.phm.0000179442.59847.27 16357554

[B15] Lannin N Lannin NA Ada L Neurorehabilitation splinting: theory and principles of clinical use NeuroRehabilitation 2011 28 1 21 28 21335674 10.3233/NRE-2011-0628

[B16] Rosenbaum P Stewart D The world health organization international classification of functioning, disability, and health: a model to guide clinical thinking, practice and research in the field of cerebral palsy Semin Pediatr Neurol 2004 11 1 5 10 15132248 10.1016/j.spen.2004.01.002

[B17] Lannin Natasha A Novak I Curtin M, Molineaux M, Supyk J Orthotics for occupational outcomes 2010 6 London: Elsevier 507 526

[B18] Louwers A Meester-Delver A Folmer K Nollet F Beelen A Immediate effect of a wrist and thumb brace on bimanual activities in children with hemiplegic cerebral palsy Dev Med Child Neurol 2011 53 4 321 326 21232053 10.1111/j.1469-8749.2010.03849.x

[B19] Elliott CM Reid SL Alderson JA Elliott BC Lycra arm splints in conjunction with goal-directed training can improve movement in children with cerebral palsy NeuroRehabilitation 2011 28 1 47 54 21335677 10.3233/NRE-2011-0631

[B20] Gauthier LV Taub E Perkins C Ortmann M Mark VW Uswatte G Remodeling the brain: plastic structural brain changes produced by different motor therapies after stroke Stroke (00392499) 2008 39 5 1520 1525 10.1161/STROKEAHA.107.502229 PMC2574634 18323492

[B21] Carr JH Shepherd RB Neurological rehabilitation: optimizing motor performance 2010 2 New York: Churchill Livingstone Elsevier

[B22] Gordon AM Schneider JA Chinnan A Charles JR Efficacy of a hand-arm bimanual intensive therapy (HABIT) in children with hemiplegic cerebral palsy: a randomized control trial Dev Med Child Neurol 2007 49 11 830 838 17979861 10.1111/j.1469-8749.2007.00830.x

[B23] Aarts PB Hartingsveldt M Anderson PG Tillaar I Burg J Geurts AC The pirate group intervention protocol: description and a case report of a modified constraint-induced movement therapy combined with bimanual training for young children with unilateral spastic cerebral palsy Occup Ther Int 2012 19 2 76 87 21751275 10.1002/oti.321

[B24] Wright FV Rosenbaum PL Goldsmith CH Law M Fehlings DL How do changes in body functions and structures, activity, and participation relate in children with cerebral palsy? Dev Med Child Neurol 2008 50 4 283 289 18312423 10.1111/j.1469-8749.2008.02037.x

[B25] Novak I Evidence to practice commentary: is more therapy better? Phys Occup Ther Pediatr 2012 32 4 383 387 23030607 10.3109/01942638.2012.726894

[B26] Sakzewski L Ziviani J Abbott DF Macdonell RA Jackson GD Boyd RN Randomized trial of constraint-induced movement therapy and bimanual training on activity outcomes for children with congenital hemiplegia Dev Med Child Neurol 2011 53 4 313 320 21401585 10.1111/j.1469-8749.2010.03859.x

[B27] Boyd RN Ziviani J Sakzewski L Miller L Bowden J Cunnington R Ware R Guzzetta A Macdonell RAL Jackson GD Abbott DF Rose S COMBIT: protocol of a randomised comparison trial of COMbined modified constraint induced movement therapy and bimanual intensive training with distributed model of standard upper limb rehabilitation in children with congenital hemiplegia BMC Neurol 2013 13 1 1 17 23809257 10.1186/1471-2377-13-68 PMC3750247

[B28] Missiuna C Mandich AD Polatajko HJ Malloy-Miller T Cognition orientation to daily occupational performance (CO-OP): part I – theoretical foundations Phys Occup Ther Pediatr 2000 20 2/3 69 81 11345513

[B29] Polatajko HJ Mandich A Enabling occupation in children: the cognitive approach to occupational performance (CO-OP) approach 2004 Ottowa, Ontario: COAT Publications ACE

[B30] Polatajko HJ Mandich AD Missiuna C Miller LT Macnab JJ Malloy-Miller T Kinsella EA Cognitive orientation to daily occupational performance (CO-OP): part III: the protocol in brief Phys Occup Ther Pediatr 2000 20 2/3 107 123 11345506

[B31] Green D Schertz M Gordon AM Moore A Schejter Margalit T Farquharson Y Ben Bashat D Weinstein M Lin J-P Fattal-Valevski A A multi-site study of functional outcomes following a themed approach to hand-arm bimanual intensive therapy for children with hemiplegia Dev Med Child Neurol 2013 55 6 527 533 23458353 10.1111/dmcn.12113

[B32] Ward A Rodger S The application of cognitive orientation to daily occupational performance (CO-OP) with children 5-7 years with developmental coordination disorder The British J of Occup Ther 2004 67 6 256 264

[B33] Polatajko HJ Mandich AD Miller LT Macnab JJ Cognitive orientation to daily occupational performance (CO-OP): part II – the evidence Phys Occup Ther Pediatr 2000 20 2/3 83 106 11345514

[B34] Rodger S Brandenburg J Cognitive orientation to (daily) occupational performance (CO-OP) with children with Asperger’s syndrome who have motor-based occupational performance goals Aust Occup Ther J 2009 56 1 41 50 20854488 10.1111/j.1440-1630.2008.00739.x

[B35] Rodger S Pham C Mitchell S Cognitive strategy use by children with Asperger’s syndrome during intervention for motor-based goals Aust Occup Ther J 2009 56 2 103 111 20854499 10.1111/j.1440-1630.2007.00719.x

[B36] Phelan S Steinke L Mandich A Exploring a cognitive intervention for children with pervasive developmental disorder Can J Occup Ther 2009 76 1 23 28 19341019 10.1177/000841740907600107

[B37] Missiuna C DeMatteo C Hanna S Mandich A Law M Mahoney W Scott L Exploring the use of cognitive intervention for children with acquired brain injury Phys Occup Ther Pediatr 2010 30 3 205 219 20608858 10.3109/01942631003761554

[B38] Cameron D Cognitive orientation to occupational performance (CO-OP): a new approach for children with cerebral palsy 2010 Chile: WFOT 10.1080/01942638.2016.1185500 27282077

[B39] Levac D Wishart L Missiuna C Wright V The application of motor learning strategies within functionally based interventions for children with neuromotor conditions Pediatr Phys Ther 2009 21 4 345 355 19923975 10.1097/PEP.0b013e3181beb09d

[B40] Valvano J Activity-focused motor interventions for children with neurological conditions Phys Occup Ther Pediatr 2004 24 1/2 79 107 15268999 10.1300/j006v24n01_04

[B41] Wilton J Casting, splinting, and physical and occupational therapy of hand deformity and dysfunction in cerebral palsy [corrected] [published erratum appears in HAND CLIN 2004 May;20(2):227] Hand Clin 2003 19 4 573 584 14596549 10.1016/s0749-0712(03)00044-1

[B42] Geerdink Y Aarts P Geurts AC Motor learning curve and long-term effectiveness of modified constraint-induced movement therapy in children with unilateral cerebral palsy: a randomized controlled trial Res Dev Disabil 2013 34 3 923 931 23291509 10.1016/j.ridd.2012.11.011

[B43] Schroder N Crabtree MJ Lyall-Watson S The effectiveness of splinting as perceived by the parents of children with juvenile idiopathic arthritis Br J Occup Ther 2002 65 2 75 80

[B44] Crompton J Imms C McCoy A Randall M Eldridge B Scoullar B Group-based task-related training for children with cerebral palsy Phys Occup Ther Pediatr 2007 27 4 43 65 18032149

[B45] Ann-Christin E Lena K-S Birgit R Eva B Marianne A Ann-Marie Ö Peter R The manual ability classification system (MACS) for children with cerebral palsy: scale development and evidence of validity and reliability Dev Med Child Neurol 2006 48 7 549 554 16780622 10.1017/S0012162206001162

[B46] Palisano R Rosenbaum P Walter S Russell D Wood E Galuppi B Development and reliability of a system to classify gross motor function in children with cerebral palsy Dev Med Child Neurol 1997 39 4 214 223 9183258 10.1111/j.1469-8749.1997.tb07414.x

[B47] House JH Gwathmey FW Fidler MO A dynamic approach to the thumb-in palm deformity in cerebral palsy J Bone Joint Surg Ame Vol 1981 63 2 216 225 7462278

[B48] Klingels K De Cock P Molenaers G Desloovere K Huenaerts C Jaspers E Feys H Upper limb motor and sensory impairments in children with hemiplegic cerebral palsy: can they be measured reliably? Disabil Rehabil 2010 32 5 409 416 20095955 10.3109/09638280903171469

[B49] Law M Baptiste S Carswell A McColl M Polatajko HJ Pollock N COPM Canadian occupational performance measure 2005 4 Ottowa, ON: CAOT Publications ACE

[B50] Cusick A McIntyre S Novak I Lannin N Lowe K A comparison of goal attainment scaling and the Canadian occupational performance measure for paediatric rehabilitation research Pediatr Rehabil 2006 9 2 149 157 16449074 10.1080/13638490500235581

[B51] Wallen MA O’Flaherty SJ Waugh M-CA Functional outcomes of intramuscular botulinum toxin type A in the upper limbs of children with cerebral palsy: a phase II trial Arch Phys Med Rehabil 2004 85 2 192 200 14966702 10.1016/j.apmr.2003.05.008

[B52] Steenbeek D Gorter JW Ketelaar M Galama K Lindeman E Responsiveness of goal attainment scaling in comparison to two standardized measures in outcome evaluation of children with cerebral palsy Clin Rehabil 2011 25 12 1128 1139 21795404 10.1177/0269215511407220

[B53] Tennant A Goal attainment scaling: current methodological challenges Dis Rehab 2007 29 20–21 1583 1588 10.1080/09638280701618828 17882728

[B54] Mathiowetz V Federman S Wiemer D Box and blocks test for manual dexterity: norms for 6-19 year olds Can J Occup Ther 1985 52 241 245

[B55] Siebers A Öberg U Skargren E The effect of modified constraint-induced movement therapy on spasticity and motor function of the affected arm in patients with chronic stroke Physiother Can 2010 62 4 388 396 21886380 10.3138/physio.62.4.388 PMC2958081

[B56] Bassini L Patel M Cooper C Pediatric hand therapy Fundamentals of hand therapy: clinical reasoning and treatment guidelines for common diagnoses of the upper extremity 2007 St Louis, Missouri: Mosby Elsevier

